# Ensemble and Pre-Training Approach for Echo State Network and Extreme Learning Machine Models

**DOI:** 10.3390/e26030215

**Published:** 2024-02-28

**Authors:** Lingyu Tang, Jun Wang, Mengyao Wang, Chunyu Zhao

**Affiliations:** 1 School of Science, Civil Aviation Flight University of China, Guanghan 618307, China; tanglingyu_cafuc@126.com; 2 Business School, Sichuan Normal University, Chengdu 610101, China; wangmychn@163.com (M.W.); zcy@sicnu.edu.cn (C.Z.)

**Keywords:** echo state network, extreme learning machine, pre-training, global random selection, ensemble training

## Abstract

The echo state network (ESN) is a recurrent neural network that has yielded state-of-the-art results in many areas owing to its rapid learning ability and the fact that the weights of input neurons and hidden neurons are fixed throughout the learning process. However, the setting procedure for initializing the ESN’s recurrent structure may lead to difficulties in designing a sound reservoir that matches a specific task. This paper proposes an improved pre-training method to adjust the model’s parameters and topology to obtain an adaptive reservoir for a given application. Two strategies, namely global random selection and ensemble training, are introduced to pre-train the randomly initialized ESN model. Specifically, particle swarm optimization is applied to optimize chosen fixed and global weight values within the network, and the reliability and stability of the pre-trained model are enhanced by employing the ensemble training strategy. In addition, we test the feasibility of the model for time series prediction on six benchmarks and two real-life datasets. The experimental results show a clear enhancement in the ESN learning results. Furthermore, the proposed global random selection and ensemble training strategies are also applied to pre-train the extreme learning machine (ELM), which has a similar training process to the ESN model. Numerical experiments are subsequently carried out on the above-mentioned eight datasets. The experimental findings consistently show that the performance of the proposed pre-trained ELM model is also improved significantly. The suggested two strategies can thus enhance the ESN and ELM models’ prediction accuracy and adaptability.

## 1. Introduction

Artificial intelligence-based prediction techniques, as a core class of forecasting methods, have been harnessed for tasks such as energy consumption estimation [1,2,3], electric load forecasting [4,5,6], wind speed forecasting [7,8,9], air pollution [10,11,12], traffic flow forecasting [13,14,15], COVID-19 forecasting [16,17,18], stock price prediction [19,20,21], natural language processing [22,23,24], and hydrological forecasting [25,26,27]. The feedforward neural network (FFNN) is a typical artificial intelligence-based prediction model showing research promise in multiple fields. A large body of empirical evidence suggests that these models perform well even when the quality of time series data is questionable. The extreme learning machine (ELM) is an emerging learning technique proposed for generalized single-hidden-layer FFNNs [28]. Different from the learning theory of conventional FFNNs, the hidden layer of a generalized single-layer feedforward network does not need to be tuned in the ELM. The method’s advantages (e.g., simplicity, interpretability, fast learning, and potential for high-quality forecasting results) have garnered close attention in the domains of medical diagnosis [29,30], signal analysis [31,32], tourism prediction [33,34], and image recognition [35,36]. However, due to a lack of recurrent or cyclic connections between neurons in the hidden layer or the output layer, an FFNN only provides a static mapping between input and output layers as information is spread throughout the network in a feedforward manner only. This constraint leads to a restricted memory and limited ability to store information. An FFNN is therefore inadequate for certain sequence tasks.

Recurrent neural networks (RNNs) are neural networks that can capture all information stored in sequence in the previous element. RNNs have feedback connections between their layers, in contrast to the FFNN model. These recurrent connections create rich dynamics and permit data flow across different layers. An RNN can accordingly make use of the information in a relatively long sequence. Specifically, RNNs perform the same tasks for every element in the sequence, with output dependent on all previous computations. These networks can hence reveal nonlinear system behavior. Because RNNs can effectively capture contextual data from a sequence, such networks have drawn wide attention and are being increasingly applied in several prediction contexts.

For example, the long short-term memory (LSTM) neural network is a popular variation of RNN-based representation learning and has been extensively adopted. Bi et al. [37] proposed LSTM networks including multivariate time series data to forecast daily visitation at tourist attractions. Qing et al. [38] devised a solar prediction scheme for hour-ahead solar irradiance prediction via an LSTM network, taking weather forecasting data into account. Abbasimehr et al. [39] constructed a demand forecasting method based on multi-layer LSTM networks. Bappy et al. [40] leveraged resampling features, LSTM cells, and an encoder–decoder network to detect image forgery. Ullah et al. [41] created a motion recognition method using a convolutional neural network and a deep bidirectional LSTM network to process video data. 

Gated recurrent units (GRUs) are a common type of gated RNN. Fanta et al. [42] deemed a novel version of a GRU, called a single-tunneled GRU for abnormality detection. Ravanelli et al. [43] revised GRUs and crafted a simplified architecture for automatic speech recognition. Zhao et al. [44] proposed local feature-based GRU networks for machine health monitoring. The attention model is an appealing prospect in the field of deep learning and has been used to process sequence-related data; this model simulates the attention mechanism of the human brain. Zheng et al. [45] created a model, correlating time-series-oriented LSTM with an attention mechanism, to study multiple tourist attractions on an hourly scale. Mou et al. [46] presented a framework for driver stress detection through multimodal fusion using attention-based deep learning techniques. Relatedly, Y. Li et al. [47] put forth an evolutionary attention-based LSTM training method with a competitive random search. By transferring shared parameters, an evolutionary attention learning approach was introduced to LSTM. Song et al. [48] suggested an attention-based LSTM network for facilitating action analysis based on skeleton data.

Although RNNs are adept at dealing with temporal information and are particularly capable of approximating arbitrary nonlinear dynamic systems with precision in theory, practitioners have always struggled to train RNNs using gradient-based approaches due to high computational costs, slow convergence, and vanishing gradients. Reservoir computing is grounded in the idea of using a large randomly and sparsely connected recurrent layer called a reservoir. This method presents an efficient alternative to gradient-based learning algorithms for designing and training RNNs in most cases [49]. The echo state network (ESN) and its variants have stimulated researchers’ interest in recent decades owing to simple model training. Like the ELM model, only the weights feeding into output neurons need to be updated in ESNs. The weights can be calculated through a simple linear regression problem, while all other weights are generated randomly and remain unchanged. ESNs have been used in various tasks. For instance, Lv et al. [50] proposed a hybrid model integrating a stacked autoencoder with echo state regression to forecast tourist flows based on search query data. Hu et al. [51] constructed a hybrid model named VMD-DE-ESN by incorporating variational mode decomposition (VMD) and differential evolution (DE) into an ESN for wind speed projections. Ma et al. [52] put forward a novel ESN approach, functional ESN, for time series classification.

It should be noticed that, while most of the literature has revealed the merits of ESNs, the efforts are still unable to clearly recognize the adaptable implications for the models. Only training hidden-output layer weights can adversely affect the training results and compromise ESNs’ performance in many use cases [53]. The network’s parameters and topology therefore are required to be adjusted to create a sound reservoir for a given application [54]. Basterrech et al. [55] have suggested the use of particle swarm optimization (PSO) to identify initial hidden–hidden weights in the ESN model. The authors modified a subset of reservoir weights and kept the rest of the weights fixed during training. Naima et al. [53] also proposed a pre-trained ESN model. They posited that, for a given ESN structure, some of the fixed non-zero weights from input-to-hidden, hidden-to-hidden, and output-to-hidden connections could be randomly selected and optimized. Then, the obtained weights would be reinjected into the network and proceed to conduct normal learning once the optimization process was complete. 

The study [53] draws our attention to two key and pertinent questions. First, is it possible to randomly select some weights for pre-training while maintaining performance consistency? In such a case, different subsets of weights for pre-training might result in distinct model performance. It is therefore important to determine ways to obtain reliable, stable performance for a given task based on a pre-trained ESN. Second, would the ESN model’s performance be improved by only taking non-zero elements as pre-training candidates? A neural network’s structure is primarily determined by the connections among network nodes, and the strength of these connections is reflected in the weight between two related nodes. Thus, updating the non-zero weights merely modifies the connection strength instead of changing the actual neural network. Network performance would be severely hampered if an initialized ESN with an unreasonable structure were generated for a specific training task. Although pre-optimization weighting could improve deficient network performance to some extent, the network structure’s innate inappropriateness would persist, as the structure of the network would not demonstrably change.

According to the questions, this paper introduces two strategies, namely ensemble training and global random selection, to improve model performance and address the above lines of inquiry. (1) The ensemble training is expected to increase performance robustness. As discussed, the fixed weights to be optimized will be randomly selected (i.e., it is unknown which weights are more important, and there is no guarantee that predominant weights will be chosen). The ensemble operates well in most situations, especially when small changes in the network structure might generate large changes in forecasting performance. (2) The global random selection strategy, which expands the selection range of pre-training weights from the non-zero range to all randomly initialized fixed weights (including zero candidates), might improve the adaptability of the network architecture. Introducing both strategies is hoped to result in a reasonable network structure with optimized weights for a specific task. 

In accordance with the two introduced strategies and the basic model proposed in [53], a new model named EN-PSO-ESN (ensemble pre-trained ESN model by PSO) is proposed. The contributions of this paper are as follows. First, the proposed model adopts a pre-training strategy and improves the network’s adaptability when facing different tasks with unique data characteristics. That is, the network has suitable presuppositions when training specific data rather than treating all circumstances generically. Second, partially fixed weight optimization reduces calculation costs while keeping the ESN’s basic properties. In addition, considering comparability, we apply the PSO algorithm for pre-training that has appeared in [53]. Third, introducing an ensemble strategy could enhance the model’s generalizability. Fourth, we expand the selection range of pre-training weights from the non-zero range to all randomly initialized weights. Some zero weights may change to non-zero weights during pre-training, while some non-zero weights might be updated. The neural network structure will be modified as a result. We can therefore transform a network with an unreasonable structure into one with a reasonable structure. In short, all randomly initialized weights that serve as pre-training candidates will enrich the basic model’s diversity for the ensemble. 

Moreover, we present a parallel study on the ELM model for a similar training process with the ESN model. The highlight of the ELM model is that input weights and biases are randomly generated, and only hidden-layer parameters should be tuned. Therefore, borrowing the premise from [53], we build a PSO-ELM model and introduce the above-discussed strategies to establish the EN-PSO-ELM model. A comparative study between PSO-ELM and EN-PSO-ELM and between PSO-ESN and EN-PSO-ESN is subsequently performed.

The remainder of this paper is organized as follows: Section 2 provides an overview of ESN, ELM, and PSO. Section 3 details the proposed EN-PSO-ESN model with ensemble training and global random selection. In Section 4, our approach is applied to eight datasets. Several simulation results confirm the efficiency of this method. Conclusions and directions for future work are outlined in Section 5.

## 2. Methodology

### 2.1. Basic Theory of Echo State Network

An ESN is a promising type of RNN that can be used to model and predict the temporal behavior of nonlinear dynamic systems [56]. The basic ESN includes an input layer, reservoir layer, and output layer. Assume that the ESN has N reservoir units, K input units, and H neurons in the output layer. At time step t, the input state matrix is given as $u\left(t\right)={\left(u_{1}\left(t\right),u_{2}\left(t\right),{\ldots ,u}_{K}\left(t\right)\right)}^{T}\in R^{K}$, the reservoir state matrix is $x\left(t\right)={\left(x\left(t\right),x_{2}\left(t\right),{\ldots ,x}_{N}\left(t\right)\right)}^{T}\in R^{N}$, and the output state matrix is $y\left(t\right)={\left(y_{1}\left(t\right),y_{2}\left(t\right),{\ldots ,y}_{H}\left(t\right)\right)}^{T}\in R^{H}$, where t=1,2,…,T and t represents the total training time step.

The reservoir states are updated as

(1) $$x\left(t+1\right)=f(W^{in}\cdot u\left(t+1\right)+W\cdot x\left(t\right)+W^{fb}\cdot y(t))$$

where f(·) is the internal activation function of the reservoir; the Sigmoid function or tanh function is usually selected, which makes the ESN have good nonlinear characteristics. $W^{in}$ is the N×K input weight matrix; W is the N×N reservoir weight matrix, which is a randomly initialized sparse matrix; $W_{fb}$ is the N×H output feedback matrix. For tasks where no output feedback is required, $W^{fb}$ is nulled. Matrices $W^{in}$, W, and $W^{fb}$ are randomly generated obeying a uniform distribution and remain unchanged during the training process.

The output layer is defined as

(2) $$y\left(t+1\right)=g(W^{out}\cdot \left[\;u\left(t+1\right);\;x\left(t+1\right)\right])$$

where g(·) is the activation fucntion of output units; [;] stands for a vertical vector (or matrix) concatenation; $W^{out}$ is an H×(K+N) matrix of output weights that need to be trained in ESN learning.

If the activation function g(·) is an identity function, the outputs from an ESN are typically linear and feedforward, Equation (2) can be written in a matrix form:

(3) $$Y=W^{out}\cdot X$$

where $Y\in R^{H\times T}$ and $X\in R^{(K+N)\times T}$ is the matrix form of $\left[u\left(t\right);\;x\left(t\right)\right]$. Then, $W^{out}$ can be represented as follows:

(4) $$W^{out}=YX^{†}$$

where $X^{†}$ is the pseudo-inverse of X.

### 2.2. Basic Theory of Extreme Learning Machine

**Figure 1:**
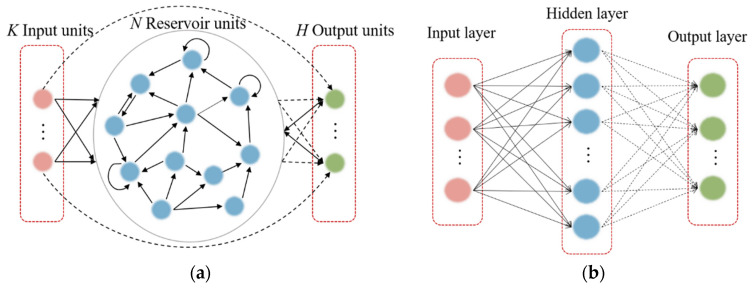
(**a**) Basic structure of an ESN model and (**b**) basic structure of an ELM model. Solid bold arrows represent fixed connections, and dashed arrows represent connections to be trained.

The ELM, proposed in [57], is a single-hidden-layer FFNN that has been broadly applied given its swift learning speed and generalization ability. A highlight of the ELM model is that input weights and biases are randomly generated, and only hidden-layer parameters must be tuned (Figure 1b). ELM learning theory maintains that these random hidden neurons can often be generated independently of the training data and application environment. The ELM therefore offers many advantages such as rapid learning and minimal manual intervention.

For T arbitrary samples $\left\{\left(u\left(t\right),y\left(t\right)\right),t=1,2,\ldots ,T\right\}$, $u(t)={\left(u_{1}\left(t\right),u_{2}\left(t\right),{\ldots ,u}_{K}\left(t\right)\right)}^{T}\in R^{K}$, $y(t)={\left(y_{1}\left(t\right),y_{2}\left(t\right),{\ldots ,y}_{H}\left(t\right)\right)}^{T}\in R^{H}$. A standard single-hidden-layer FFNN with L hidden nodes and activation function f(·) is modeled as

(5) $$H\left(t\right)=f\left(W^{in}\cdot u\left(t\right)+\beta \right),t=1,2,\ldots ,T$$

where $H\left(t\right)$ is the output of the hidden layer with L×1 dimensions corresponding to the input of the *t*-th sample; $W^{in}$ is a L×K input weight matrix; β is a L×1 bias vector; $W^{in}\cdot u(t)$ denotes the inner product of $W^{in}$ and u(t).

For the total *T* samples, Equation (5) can be written in a matrix form:

(6) $$H=f\left(W^{in}\cdot u+\beta \right)$$

where H is the output of the hidden layer with L×T dimensions; u is the input matrix of all samples. Then, the output of the ELM network Y can be expressed as

(7) $$Y=W^{out}\cdot H$$

where Y is an H×T matrix and $W^{out}$ is an H×L output weight matrix that connects the hidden layer and the output layer; $W^{out}$ can be represented as

(8) $$W^{out}=YH^{†}$$

where $H^{†}$ is the pseudo-inverse of H.

Through the above description, a brief comparison can be made between ESNs and ELMs. ESNs are based on reservoir computing, which involves a randomly connected network with fixed weights that captures the temporal dynamics of the input data. Conversely, ELMs belong to feedforward neural networks with random hidden layer weights and a linear output layer. Despite differences in network structure, both ESN and ELM fix other parameters and only train the weights of the output layer during the training process. This makes both ESN and ELM networks capable of handling large-scale datasets and well suited for real-time applications with high efficiency. Therefore, in our papers, we have conducted corresponding research on both networks.

### 2.3. Basic Particle Swarm Optimization

**Figure 2:**
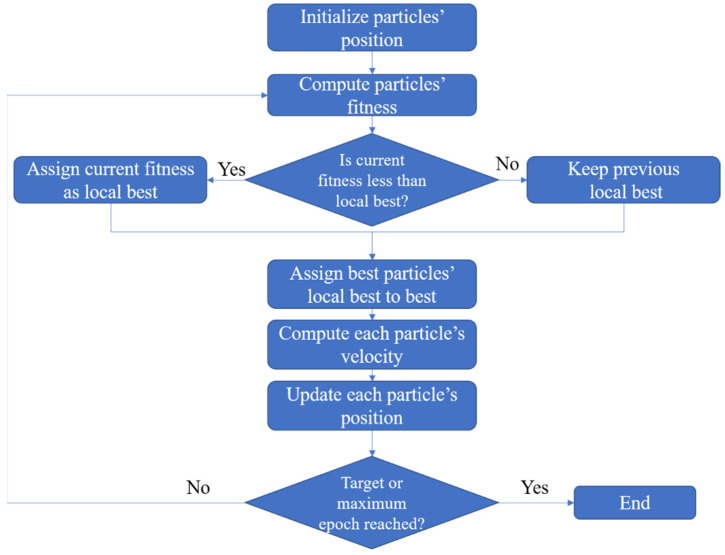
Flowchart of basic PSO algorithm.

Kennedy and Eberhart proposed PSO in 1995; it is an exemplar of swarm intelligence technology, inspired by birds’ foraging behavior [57]. The seminal PSO paper described a population-based optimization algorithm for searching for an optimum in a search space: a group of particles is randomly initiated in hyperspace, and then each particle moves in the hyperspace at a dynamically adjusted velocity that is determined by its own best experience as well as the entire population’s best experience. Mathematically, the PSO algorithm can be briefly described as follows:

To solve an optimization problem in a *d*-dimensional space, N particles are considered as initial solutions. The location of the *i*th particle in a *d*-dimensional space can be represented as $X_{i}=\left(x_{i1},x_{i1},\ldots ,x_{id}\right),$ and $V_{i}=(v_{i1},v_{i1},\ldots ,v_{id})$ represents the displacement of each iteration. Particles update themselves using two extremes: one is the optimal solution $P_{i}=\left(p_{i1},p_{id},\ldots ,p_{id}\;\right),$ calculated by itself, and the other is the optimal solution $P_{g}$, calculated by the global group. The iteration formula is as follows:

(9) $$v_{id}=\omega \times v_{id}+c_{1}r_{1}\times \left(p_{id}-x_{id}\right)+c_{2}r_{2}(p_{g}-x_{id})$$

(10) $$x_{id}=x_{id}+v_{id}$$

where ω denotes the inertia weight, $c_{1}$ and $c_{2}$ denote acceleration factors, $r_{1}$ and $r_{2}$ are random numbers evenly distributed in the range [0, 1], $\omega \times v_{id}$ represents the effect of the previous speed on the current speed, $c_{1}r_{1}\times \left(p_{id}-x_{id}\right)$ allows particles to adjust according to their optimal position, and $c_{2}r_{2}(p_{g}-x_{id})$ allows particles to adjust through the optimum of the population. The flowchart of the basic PSO algorithm is depicted in Figure 2.

We chose PSO for selected weight pre-training considering two considerations. First, PSO is an important means of addressing optimization; the algorithm has been applied in a range of fields since its conception, and a growing number of practical problems have been solved using PSO or its variants. The algorithm therefore possesses a strong ability to obtain the global optimum. Second, the model proposed in this study was inspired by [53], which adopted the PSO approach for pre-training. To make a fair comparison with the model put forth in [53] and to verify these models’ performance, using the same optimization technique is imperative. In addition, the initial values of the parameters $c_{1},\;c_{2}$, and ω are also initialized as 1.193, 1.193, and 0.721, respectively, in this study, and then at each iteration, $c_{1}$ and $c_{2}$ take their values from a uniform distribution between 0 and their initial values as well.

## 3. Proposed EN-PSO-ESN Model

### 3.1. Motivation

As mentioned, [53] proposed a pre-training strategy to optimize some non-zero weights randomly collected in matrices $W^{in}$, W, and $W^{fb}$ before ESN learning to provide weights suited to the target task. Two concerns apply in this case: (1) how to maintain the robustness of the model’s performance and (2) which selection strategy can most effectively boost the model’s performance—only choosing non-zero weights as candidates, or considering both non-zero and zero weights?

Regarding the first issue, selecting some weights randomly for pre-training is a matter of trial and error to enhance network performance. The model may exhibit satisfactory performance with a randomly chosen set of weights; however, there is no guarantee that the model will suit the target application in another random selection. In essence, for any given task, it would be impossible to forecast which connection weights will significantly affect network performance. Similarly, it is not possible to project which weights have more potential for optimization. Ensuring the pre-trained ESN’s reliable and stable performance by randomly choosing fixed weights is correspondingly important. 

For the second question, the connections among network nodes determine the neural network’s structure. The strength of connections is reflected by the weight between two related nodes. Thus, simply updating non-zero weights does not alter the network structure and only affects connection strength. Choosing non-zero elements for pre-training means that the network’s optimization is based on a fixed and pre-determined structure. The initialization creates unsuitable input-to-hidden and hidden-to-hidden links, and the basic network contains innate structural deficiencies. Weight pre-optimization can improve the network’s performance; however, the inherent inappropriateness of its structure persists.

**Figure 3:**
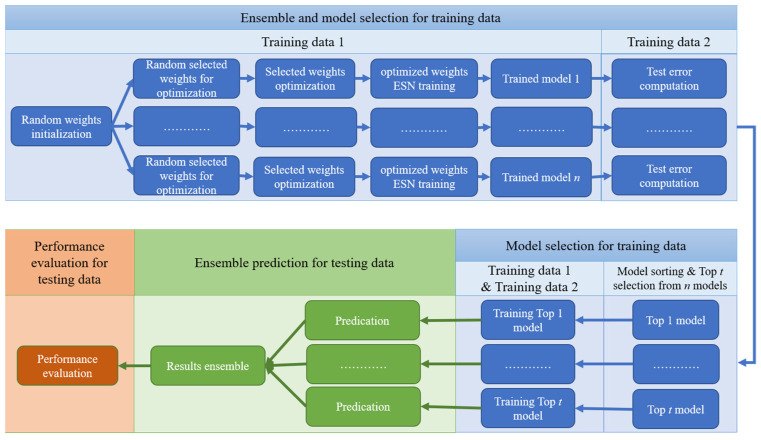
Flowchart of EN-PSO-ESN model.

Based on [53] and the above two issues, this paper introduces two strategies (i.e., ensemble training and global random selection) to the PSO-ESN model. A new model, EN-PSO-ESN, is described in this section. An ensemble training strategy is applied to increase the robustness of network performance. The global random selection strategy expands the range of possible pre-training weights from the non-zero range to all randomly initialized fixed weights. Figure 3 illustrates a flowchart of the overall process for the proposed model; details are provided in Section 3.2.

### 3.2. Main Steps of EN-PSO-ESN Model

As Figure 3 indicates, the proposed model comprises three main stages: (1) model training and selection (blue block); (2) ensemble prediction (green block); and (3) performance evaluation (orange block). The first two stages are the core tenets of the proposed approach. The full process can be divided into several steps.

**Step 1**. **Construct *n* independent basic ESN models**. *n* independent basic ESN models are initialized. Each model has the same amount of input, reservoir units, and output as well as the same connection rate for reservoir units. Then, the input-to-hidden and hidden-to-hidden link weights are randomly initialized separately.

**Step 2**. **Select random weights**. For each of the n independent initialized ESN models, the weights needing to be pre-optimized are randomly chosen. These weights include some from the input-to-hidden, hidden-to-hidden, and output-to-hidden connections and cover all elements belonging to the three weight matrices $W^{in}$, W, and $W^{fb}$, including zero and non-zero elements. Suppose parameters α, β, and γ are the chosen rates for the three weight matrices. Then, the total number of pre-training weights can be represented as $\alpha \phi (W^{in})+\beta \phi (W)+\gamma \phi (W^{fb})$, where ϕ(⋅) denotes the number of elements in a matrix, and the parameters α, β, and γ belong to the interval $\left[0,1\right].$ It is advisable to assign these parameters small values to choose an array of weights to be pre-trained; just a few selected weights can preserve the model’s specificity while controlling the computational cost for weight optimization. 

**Step 3**. **Optimize selected weights.** Because of the advantages of PSO and to offer a fair comparison with [53], PSO is chosen as the optimization technique. The training set is divided into two parts, namely Set 1 and Set 2. Set 1 is obtained by sampling the original training set: for the given sampling ratio s%, s% training samples are sampled to establish Set 1. Set 2 contains the remaining data from Set 1. If the size of the training set is insufficient, then sampling can be performed with the replacement method to ensure that Set 1 includes an adequate number of samples. 

**Figure 4:**
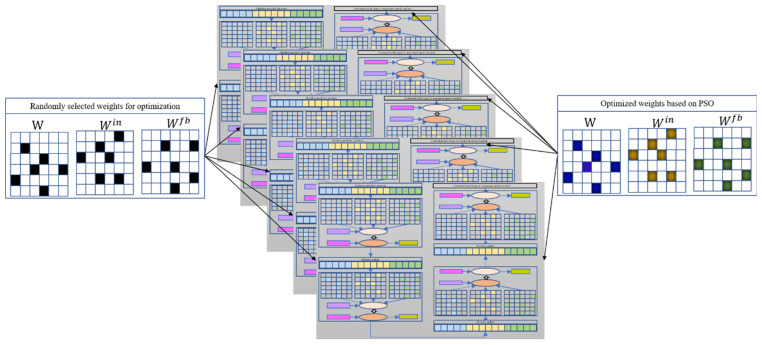
Pre-training framework of *N* basic ESNs based on PSO algorithm.

**Figure 5:**
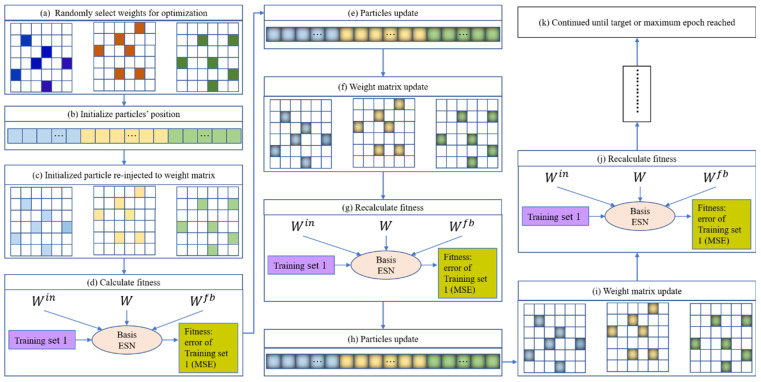
Iteration procedure of a given particle.

Consider a basic ESN model as an example of optimizing randomly chosen weights. Once the weights that need to be pre-optimized have been randomly selected, the total number of pre-training weights and their locations in the corresponding matrix are determined. PSO can then be used to optimize the selected weights; the optimization diagram is shown in Figure 4. Here, each particle represents a candidate solution vector (as pictured in Figure 5b), and the particle length represents the number of weights to be optimized. For a given initialized particle, the corresponding solution vector is injected into the three weight matrices $W^{in}$, W, and $W^{fb}$ (in Figure 5c). The basic ESN model is next trained based on Set 1 (Figure 5d). After training the basic ESN model, the training error (i.e., the root mean square error (RMSE); see Equation (11)) of the model is set as the fitness value to measure the current particle’s performance. If the fitness value does not meet the stop criterion or if the iteration epoch has been reached, then the particle is updated based on Equations (9) and (10). The updated particle is next reinjected into its corresponding position in the three weight matrices. Then, based on Set 1, the basic ESN model is re-trained, and the new fitness value is obtained. These steps are repeated until the target or maximum iteration is reached. The global best solution can be realized based on the best solution vector of each particle. Namely, the weight matrices are learned, and the basic ESN model is pre-trained. The *n* trained ESN model can be derived via the parallel optimization process in Figure 4. 

The training framework for the EN-PSO-ELM model is the same as for the EN-PSO-ESN model. The canonical ELM model is a single-layer FFNN; as such, only two weight matrices ${(W}^{in}$ and $W^{out})$ are involved, where $W^{in}$ is the input-to-hidden connection and $W^{out}$ is the hidden-to-output connection. Some weights in the sparse matrix $W^{in}$ will be chosen for pre-training. 

**Step 4. Evaluate and select a model**. For the pre-trained n ESN models, Set 2 is taken as validation data and fed into these models to evaluate their performance (i.e., the RMSE). The n models are then sorted by RMSE in ascending order. The top t models are finally chosen in line with practical application problems and used to constitute an ensemble model. 

**Step 5. Re-train the top *t* models**. To ensure that the pre-trained models can accurately capture data patterns, all training datasets, including Sets 1 and 2, are applied to re-train the selected models independently. 

**Step 6. Conduct independent and integrated prediction**. Next, corresponding prediction results are respectively derived from *t* trained models. Different integration strategies are used to obtain the final integrated prediction results, such as the optimistic choice for the largest prediction result, the negative choice for the smallest result, and the neutral choice for a weighted average result. 

**Step 7. Assess performance**. To evaluate the prediction accuracy of the proposed approach, different performance evaluation criteria (e.g., root mean square error (RMSE), relative mean absolute error (RMAE), mean absolute percentage error (MAPE), root mean square percentage error (RMSPE), and directional accuracy (DA); see Equations (11)–(15)) can be used to measure prediction errors in the test data.

## 4. Experiments and Results

In this section, the forecasting ability of the proposed model is tested against that of several benchmark models. The data description, performance evaluation criteria, and benchmarks are presented in Section 4.1. Experiments for six synthetic benchmark time series predictions are summarized in Section 4.2, with experiments for the two real benchmark time series predictions appearing in Section 4.3. Experimental results are analyzed respectively. An additional experiment performed to further illustrate the proposed model’s effectiveness is presented in Section 4.4.

### 4.1. Datasets, Performance Evaluation Criteria, and Benchmarks

Six artificial datasets and two real-life datasets were chosen in this study to demonstrate improvements attributable to the proposed model. The six artificial datasets include two Mackey and Glass time series with different parameters, two nonlinear auto-regressive moving averages with different parameters, and the Henon attractor and Lorenz attractor data series (see Equations (16)–(19)). These datasets have been drawn from [53] to facilitate a fair comparison and a reliable demonstration (i.e., that our proposed model leads to improvements). Furthermore, two real-life datasets—the air quality (AQ) dataset and airfoil self-noise (ASN) dataset—are applied; both were acquired from the Machine Learning Repository at the University of California, Irvine (UCI) [58].

To evaluate models’ forecasting performance in terms of directional prediction and level prediction, five indicators (i.e., RMSE, RMAE, MAPE, RMSPE, and DA) that have been frequently used in recent years were selected. y(t) and $\hat{y}(t)$ stand for the actual value and predicted value, respectively; $a\left(t\right)=1$, if $(y\left(t+1\right)-y(t))\left(\hat{y}\left(t+1\right)-\hat{y}(t)\right)\geq 0$, or $a\left(t\right)=0$ otherwise. N is the size of predictions. For the first four indicators, smaller index values represent higher forecasting accuracy; larger index values indicate higher forecasting accuracy for DA.

(11) $$RMSE=\sqrt{\frac{1}{N}\sum_{t=1}^{N}{\left(y\left(t\right)-\hat{y}(t)\right)}^{2}}$$

(12) $$RMAE=\frac{\sum_{t=1}^{N}\left|y\left(t\right)-\hat{y}(t)\right|}{\sum_{t=1}^{N}y(t)}$$

(13) $$MAPE=\frac{1}{N}\sum_{t=1}^{N}\left|\frac{y\left(t\right)-\hat{y}(t)}{y(t)}\right|$$

(14) $$RMSPE=\sqrt{\frac{1}{N}\sum_{t=1}^{N}{\left(\frac{y\left(t\right)-\hat{y}\left(t\right)}{y\left(t\right)}\right)}^{2}}$$

(15) $$DA=\frac{1}{N}\sum_{t=1}^{N}a(t)\times 100\%$$

Two groups of models (Table 1) were compared to test the effectiveness of the proposed strategies.

Regarding the first group—ESN-based models—the canonical ESN model serves as the foundation for the PSO-ESN model proposed in [53] and the model proposed in this study. The basic ESN model was thus taken as a benchmark. Additionally, because the proposed model was derived from the PSO-ESN model, the PSO-ESN model should be selected. 

For the second group, as noted, the ELM model shares similarities in model construction and training with the canonical ESN. Applying the two proposed strategies to the ELM-based model can further verify their effectiveness. The ELM-PSO model, in line with [53], was constructed accordingly. The corresponding EN-PSO-ELM model was established by applying ensemble training and global random selection to the PSO-ELM model.

### 4.2. Experiment for Synthetic Benchmark Time Series Prediction

**(1)**  **Mackey and Glass dataset**

Mackey and Glass (MG) time series possess chaotic nonlinear behavior, rendering it difficult to identify underlying patterns from the training samples. The dynamic predictive system and corresponding parameters are described in Equation (16):

(16) $$\dot{x(t)}=\frac{ax(t-\tau )}{1+x^{c}(t)}-bx(t)$$

where a=0.2, b=0.1, c=10. According to the dynamic equation and the different values of τ (τ=17 and τ=30), two series, MG(1) and MG(2), respectively, were generated. Each of the series contains 1000 samples, with 800 samples used as a training dataset and 200 samples used for testing. 

The above-mentioned six models were applied to MG(1) and MG(2). Some hyperparameters should be defined before model training (e.g., hidden neuron number, selection rate of the weight matrix, ensemble number). Detailed parameter information is listed in Table 2. The running times, set to 50, indicate that every model is repeated 50 times to derive its average performance. The hidden neuron number indicates the number of neurons in the hidden layer of the ELM model and the reservoir size for the ESN model. The parameters α and β denote the selection ratios of the weight elements for pre-optimization in the weight matrices $W^{in}$ and W; the feedback weight is not considered in this study. For ELM and ELM-based models, only some of the weight elements in the weight matrix $W^{in}$ are pre-trained. The parameter β is therefore not involved. The ensemble number is only for the ensemble model. The leak rate reflects the memory capacity of ESN-based models, and the collection rate of the reservoir indicates the sparsity of the ESN model.

**Figure 6:**
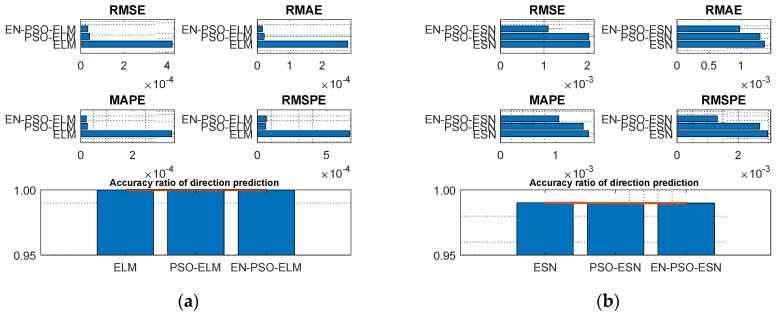
Visual representation of prediction performance for six models (MG(1) dataset). (**a**) The comparison of the average measure indices of the ELM-based models. (**b**) The comparison of the average measure indices of the ESN-based models.

The prediction results for the six models are displayed in Table 3. In general, ensemble training and global random selection substantially influenced both the ELM model and the ESN model. Taking the ELM-based model as an example, the proposed EN-PSO-ELM model was superior to the PSO-ELM model (see Figure 6a). The canonical ELM model showed a strong prediction effect for the two datasets but performed worse than the two improved models. The same conclusion applied to the ESN-based models (see Figure 6b). 

**Figure 7:**
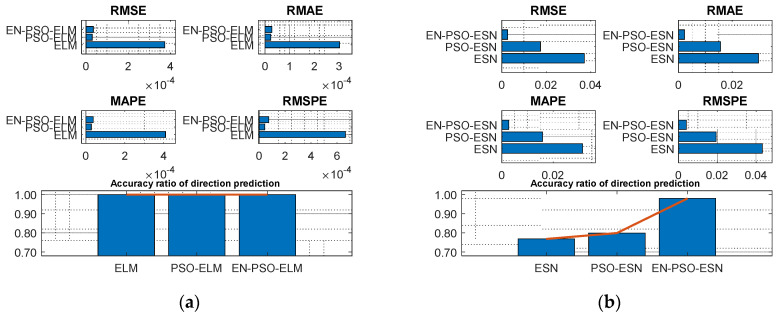
Visual representation of prediction performance for six models (MG(2) dataset). (**a**) The comparison of the average measure indices of the ELM-based models. (**b**) The comparison of the average measure indices of the ESN-based models.

For the MG(2) dataset, the EN-PSO-ELM model’s performance did not surpass all ELM-based models; it was slightly inferior to the PSO-ELM model. The accuracy of the directional prediction was identical to that of the best model, reaching 100%. Although the other four measurement indices slightly exceeded the best ones, all four indices maintained the same prediction accuracy level as the best-performing model (see Figure 7a,b).

**(2)**  **Nonlinear auto-regressive moving average dataset**

The nonlinear auto-regressive moving average (NARMA) is a popular and hard predictive time series characterized by highly chaotic behavior. The dynamics of this dataset are summarized in Equation (17):

(17) $$y\left(t+1\right)=c_{1}y\left(t\right)+c_{2}y\left(t\right)+\sum_{i=1}^{k}y(t-i)+c_{3}x\left(t-\left(k-1\right)\right)x\left(t\right)+c_{4}$$

For the given parameters $c_{1}=0.3$, $c_{2}=0.05$, $c_{3}=1.5$, $c_{4}=0.1$, *k* = 6, and *k* = 10, two series, NARMA(1) and NARMA(2), were generated. Each of the data series contained 1000 samples: 800 for training and 200 for testing.

Using the same hyperparameters as in Table 2, we derived the prediction results for NARMA(1) and NARMA(2) with different lag orders. The performance evaluations are displayed in Table 4.

**Figure 8:**
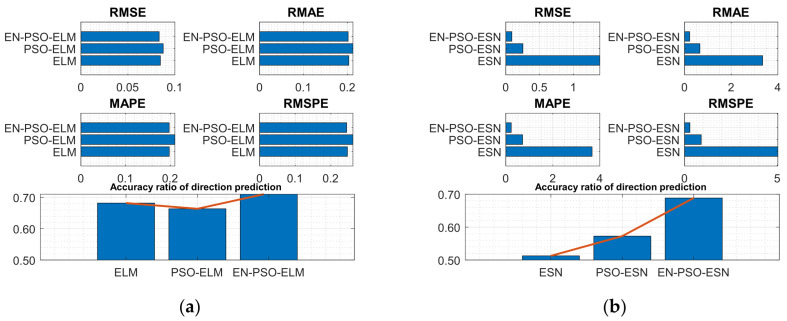
Visual representation of prediction performance for six models (NARMA(1) dataset). (**a**) The comparison of the average measure indices of the ELM-based models. (**b**) The comparison of the average measure indices of the ESN-based models.

**Figure 9:**
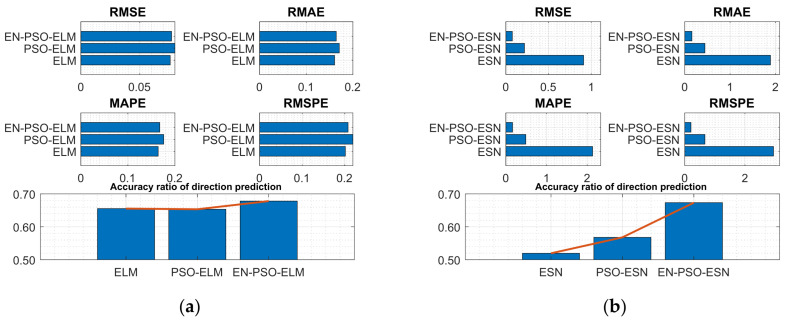
Visual representation of prediction performance for six models (NARMA(2) dataset). (**a**) The comparison of the average measure indices of the ELM-based models. (**b**) The comparison of the average measure indices of the ESN-based models.

Table 4 reveals a similar conclusion to that revealed in Table 3; that is, the proposed EN-PSO model was superior to the PSO-based model and the canonical model, including the ELM-based and ESN-based models. Yet the PSO-ELM model’s performance also warrants a note: this model was inferior to the EN-PSO-ELM model and performed worse than the canonical ELM model for both the NARMA(1) and NARMA(2) datasets (see Figure 8 and Figure 9).

**(3)**  **The Henon attractor and Lorenz attractor data series**

The Henon attractor and Lorenz attractor data series are dynamic systems frequently mentioned in relevant literature. The Henon map is a discrete-time dynamic attractor; Lorenz is a set of differential equations describing a fluid motion between a hot and a cool surface. These datasets can be generated by the corresponding Equations (18) and (19). As before, each series contained 1000 samples (800 for training and 200 for testing).

(18) $$\left\{\begin{array}{c} x\left(t+1\right)=y\left(t\right)-ax^{2}\left(t\right)+1,\\ \begin{array}{c} y\left(t+1\right)=bx\left(t\right),\\ a=1.4,\;\;b=0.3. \end{array} \end{array}\right.$$

(19) $$\left\{\begin{array}{c} \dot{x}=a(y-x)\\ \begin{array}{c} \dot{y}=-y-xz+rx\\ \begin{array}{c} \dot{z}=xy-bz\\ a=10,\;b=28,c=\frac{8}{3} \end{array} \end{array} \end{array}\right.$$

**Figure 10:**
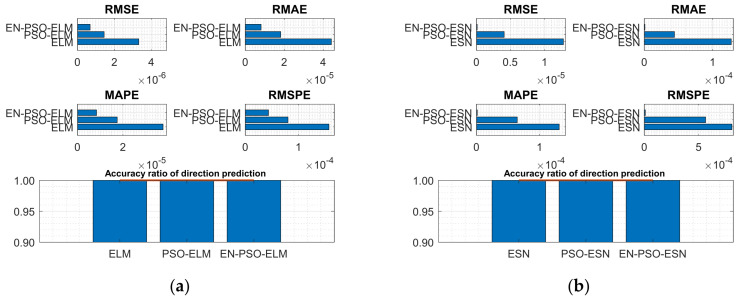
Visual representation of prediction performance for six models (Henon attractor dataset). (**a**) The comparison of the average measure indices of the ELM-based models. (**b**) The comparison of the average measure indices of the ESN-based models.

**Figure 11:**
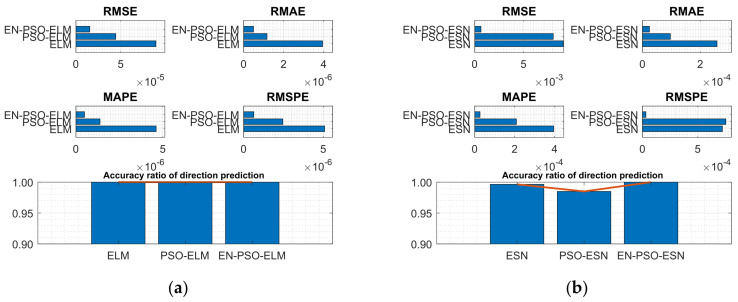
Visual representation of prediction performance for six models (Lorenz attractor dataset). (**a**) The comparison of the average measure indices of the ELM-based models. (**b**) The comparison of the average measure indices of the ESN-based models.

Experiments were conducted on both series. The associated performance measurement indices for the six models are listed in Table 5, mirroring the findings of prior experiments: the proposed EN-PSO-based model consistently performed best, followed by the PSO-based model (see Figure 10 and Figure 11).

### 4.3. Experiment for Real Benchmark Time Series Prediction

To further demonstrate the proposed model’s forecasting performance, two real-life datasets (an AQ dataset and an ASN dataset) were obtained from the UCI Machine Learning Repository. Several measurement indices, including RMSE, RMAE, MAPE, RMSPE, and DA, were used to compare the models’ forecasting accuracy. To eliminate fluctuations in forecasting performance, every model was run 50 times for both data series; the mean values of these 50 indices were calculated. Table 6 and Table 7 indicate the performance of different benchmarks.

#### 4.3.1. Air Quality (AQ) Dataset

**Figure 12:**
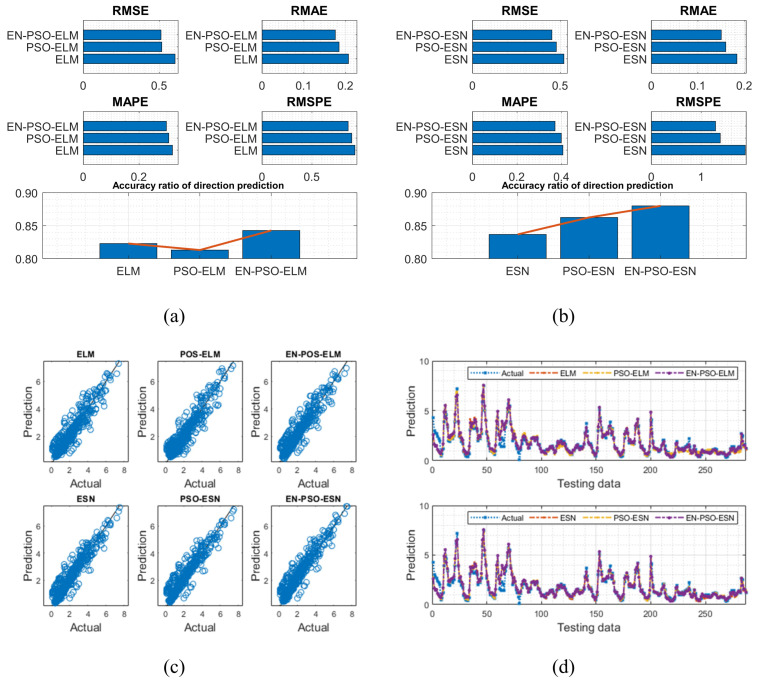
Visual representation of prediction results for six models (AQ dataset). (**a**) The comparison of the average measure indices of the ELM-based models. (**b**) The comparison of the average measure indices of the ESN-based models. (**c**) The fitting performances on the test data. (**d**) The fitting results on the test data.

The AQ dataset contained 9358 instances of hourly averaged responses from an array of five metal oxide chemical sensors in the field in a significantly polluted area of an Italian city, collected from March 2004 to February 2005. The dataset included 13 attributes (e.g., temperature, true hourly averaged NO_2_ concentration in microg/m^3^) in addition to date and time. The true hourly averaged CO concentration in mg/m^3^ was taken as the target for prediction in this work. After a preprocessing procedure was performed to exclude missing values, 90% of the data were used for training with 10% reserved for testing. The results are listed in Table 6 and are illustrated in Figure 12.

#### 4.3.2. Airfoil Self-Noise (ASN) Dataset

**Figure 13:**
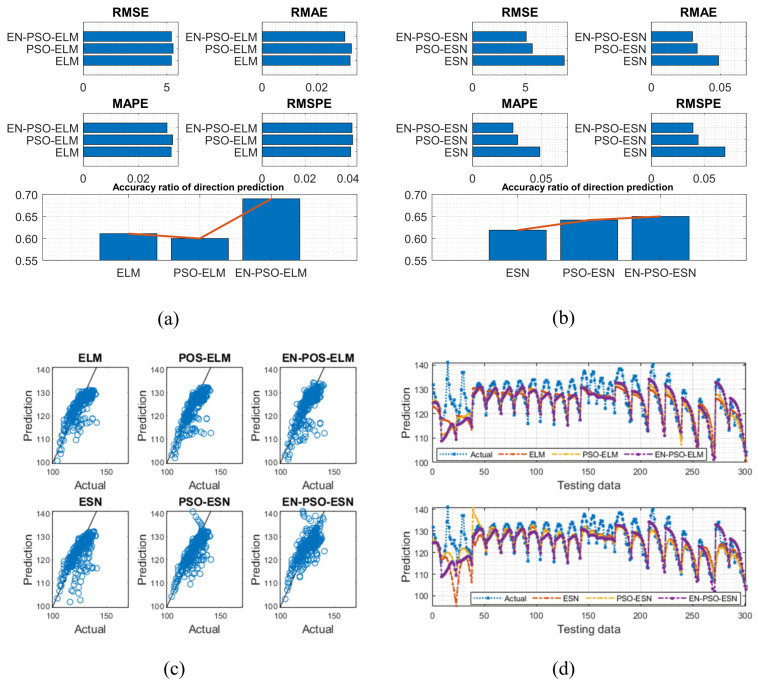
Visual representation of prediction results for six models (ASN dataset). (**a**) The comparison of the average measure indices of the ELM-based models. (**b**) The comparison of the average measure indices of the ESN-based models. (**c**) The fitting performances on the test data. (**d**) The fitting results on the test data.

The ASN dataset included five features: (a) frequency in hertz; (b) angle of attack in degrees; (c) chord length in meters; (d) free-stream velocity in meters per second; and (e) suction side displacement thickness in meters. Scaled sound pressure level was the sole output variable. The testing results are listed in Table 7 and are visually depicted in Figure 13.

Based on the eight experiments presented above, the following conclusions can be drawn: First, like findings for the six synthetic benchmark time series, the prediction results clearly showed that the proposed EN-PSO-based models’ accuracy surpassed that of POS-based and canonical models with smaller standard deviations. Taking the AQ dataset for instance, the ensemble training and global random selection strategies effectively improved forecasting accuracy whether using the ESN-based or ELM-based model. More precisely, for the EN-PSO-ELM model, the first four measurement indices respectively declined by 15.3464%, 15.2731%, 6.8253%, and 7.2051%; the accuracy of direction prediction increased by 2.4060% compared with the canonical ELM model. The first four prediction error indices decreased by 1.0844%, 4.7800%, 2.7451%, and 3.9515% compared with the PSO-ELM model and rose by 3.6654% for direction prediction, respectively. The conclusions remained consistent for the EN-PSO-ESN model, such that this model’s prediction accuracy also rose considerably and maintained a minimum standard deviation. In detail, compared with the canonical ESN model, the error indices (i.e., RMSE, RMAE, MAPE, and RMSPE) declined by 13.0744%, 18.2907%, 8.7771%, and 31.5504%, respectively. The EN-PSO-ESN model also performed better than the PSO-ESN model. Furthermore, overall, both the PSO-ELM model and the PSO-ESN model appeared superior to the canonical ELM and ESN models. 

Second, although the PSO-ELM model and PSO-ESN model each appeared superior to the canonical ELM and ESN models in most situations, the PSO-ELM model did not always outperform canonical models. For example, in terms of the ASN dataset, the PSO-ELM model performed worse than the canonical ELM model. The first four measurement indices exceeded those of the canonical ELM model by 1.9824%, 1.5625%, 1.5773%, and 2.1951%, respectively; the accuracy of direction prediction was 1.8012% lower than that for the ELM model (see Table 7). Similar findings emerged for prior experiments: as shown in Table 4, the PSO-ELM model’s performance was inferior to that of the canonical ELM for the NARMA(1) and NARMA(2) datasets. We carried out another experiment on the ASN dataset (see the next subsection) to explore why this phenomenon might have occurred. 

### 4.4. Additional Experiment

This section presents an additional experiment on the ASN dataset, focusing on a comparison between the canonical ELM model and the PSO-ELM model. This experiment was intended to uncover (a) why the PSO-ELM model did not necessarily maintain superiority over the canonical ELM model and (b) why the EN-PSO-ELM model maintained sound performance with the above eight datasets. Four models (see Table 8) were considered.

To evaluate the average performance of the above four models, we first generated 100 basic canonical ELM models for training. Put simply, according to the datasets’ input and output, 100 weight matrices $W^{in}$ were randomly generated. One hundred prediction results and corresponding measurement indices were obtained for the ASN dataset based on the basic ELM models. 

Next, the pre-training strategy was applied to 100 initialized ELM models, and 100 corresponding PSO-ELM(I), PSO-ELM(II), and EN-PSO-ELM models were derived. For the PSO-ELM(I) model, some of the non-zero weights (equal to 10% of all weight elements) were randomly selected for pre-training. The same proportion was randomly selected for the PSO-ELM(II) and EN-PSO-ELM models but covered both non-zero and zero elements. The pre-optimization and network training were thus identical to the previous experiments.

**Figure 14:**
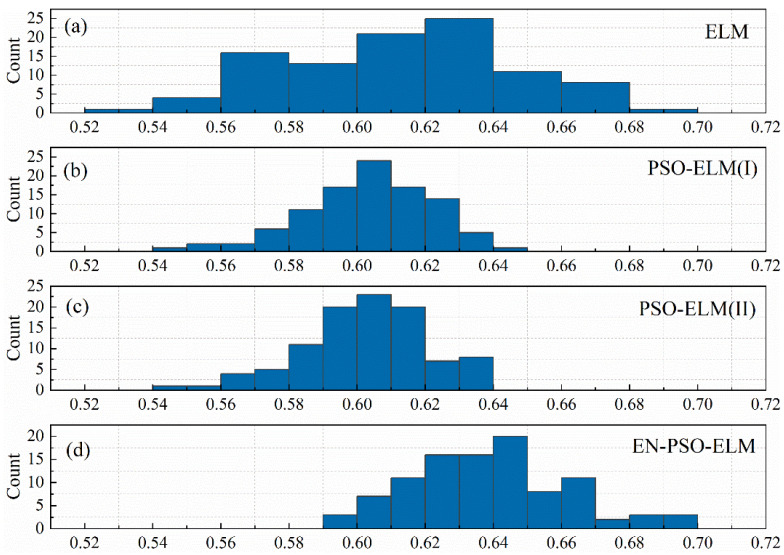
The DA distribution for four models. (**a**) The distribution of DA values for 100 initialized canonical ELM models. (**b**) The DA distribution for 100 PSO-ELM(I) models. (**c**) The DA distribution for 100 PSO-ELM(II) models. (**d**) The DA distribution for ensemble PSO-based ELM models.

To discern the performance of the compared models, DA is taken as a sample metric here to evaluate the prediction results. All models were derived from the 100 initialized basic model as indicated; doing so enabled us to observe the DA distribution and identify performance discrepancies. Figure 14 displays the DA distribution of the canonical ELM models, PSO-based ELM models (I), PSO-based ELM models (II), and ensemble PSO-based ELM models. Figure 14a, showing the DA distribution of the initialized 100 canonical ELM models, reveals that DA was distributed over a large interval (from 55% to 69%). Figure 14b,c contain the 100 DA distributions of the PSO-ELM(I) models and PSO-ELM(II) models, respectively. Both figures are similar in that the distribution is more concentrated than that in the canonical ELM models. This phenomenon suggests that the pre-trained algorithm can improve the stability of prediction performance to a certain extent. DA was also concentrated within a relatively small range in both instances—from 54% to 65% and from 56% to 64%, respectively. However, the largest DA of the two models was less than 65%. On the contrary, the largest DA of the canonical ELM models reached 69%. In sum, for the ASN dataset, the pre-training strategy seemed useful for boosting the model’s stability but had a less pronounced impact on prediction accuracy. This relatively lower improvement may explain why the PSO-ELM model performed slightly worse than the canonical ELM models. Meanwhile, for the ASN dataset, although we expanded the pre-training candidates from non-zero elements to all elements, prediction accuracy demonstrated no significant increase. Lastly, compared with Figure 14b,c, the DA distribution in Figure 14d clearly shifted to the right. The prediction accuracy therefore exhibited a significant improvement. The AD distribution in Figure 14d also maintained a relative concentration; that is, because the EN-PSO-ELM model applied the pre-training and ensemble strategy, both the prediction accuracy and robustness were elevated. 

## 5. Conclusions

In this work, we proposed two strategies—global random selection and ensemble training—for ESN and ELM models in order to pre-train the initialized weight parameters and the network topology to improve the models’ adaptability. Global random selection expands the range of chosen pre-training weights from non-zero weights to all randomly initialized weights. This characteristic enriches the basic models’ diversity. We also adopted ensemble training to enhance the pre-trained models’ reliability and stability. To ensure a fair comparison between the proposed model and the one from [53], PSO was applied as an optimization technique to realize weight pre-training. The proposed approach was then tested for time series prediction based on six benchmarks and two real-life datasets. The experimental results highlighted notable enhancements in ESN and ELM learning results. 

The experiments further show that, although the PSO-ELM model and the PSO-ESN model seemed superior to the canonical ELM and ESN models in most cases, the PSO-ELM model is not consistently superior to the canonical models. For example, the PSO-ELM model performs worse than the canonical ELM model on the ASN dataset and NARMA dataset. To investigate this outcome in greater depth, we performed a subsequent experiment with the ELM model using the ASN dataset. The four models’ DA distributions (i.e., ELM, PSO-ELM[I], PSO-ELM[II], and EN-PSO-ELM) reveal several interesting patterns. For example, with the ASN dataset, the pre-trained algorithm that only includes non-zero elements for pre-training may lead to more stable prediction performance but not better prediction accuracy. Global selection could further enhance the model’s robustness but had little effect on prediction accuracy. Yet introducing ensemble training significantly increases prediction accuracy, underscoring that these two strategies can jointly promote models’ prediction accuracy and robustness.

In conclusion, it is essential to acknowledge the limitations of this study, specifically the absence of inter-model comparisons. Our study originally intended to conduct two types of experiments: intra-model comparisons (ELM vs. EN-PSO-ELM and ESN vs. EN-PSO-ESN) and inter-model comparisons (EN-PSO-ELM vs. EN-PSO-ESN). Given that the ESN is a recurrent neural network and the ELM is a feedforward neural network, we hypothesized that EN-PSO-ESN would likely outperform EN-PSO-ELM. However, the experimental results based on the applied datasets did not support this assumption. Instead, EN-PSO-ELM performed better in some tasks, while EN-PSO-ESN excelled in others. We speculate that this outcome may be linked to two factors: (1) the initialization process of the ESN, where hyperparameters like the leaking rate, input scaling, spectral radius, and sparse degree could impact performance, and (2) the intrinsic characteristics of the dataset itself. Future work will focus on determining under which circumstances each model excels. Despite these limitations, the two proposed strategies in this study—global random selection and ensemble training—offer valuable insights into model pre-training, enhancing models’ adaptability across diverse task situations.

## Figures and Tables

**Table 1:** Models compared in this study.

	ESN-Based Models	ELM-Based Models
Compared Models	(1) Canonical ESN	(1) Canonical ELM
	(2) PSO-ESN	(2) PSO-ELM
	(3) EN-PSO-ESN	(3) EN-PSO-ELM

**Table 2:** Parameter settings for model comparison.

	Running Times	Hidden Neuron Number	*α*	*β*	Ensemble Number	Leak Rate	The Collection Rate of the Reservoir
ELM	50	100	\	\	\	\	\
PSO-ELM	50	100	0.1	\	\	\	\
EN-PSO-ELM	50	100	0.1	\	50	\	\
Canonical ESN	50	100	0.1	0.01	\	0.3	0.01
PSO-ESN	50	100	0.1	0.01	\	0.3	0.01
EN-PSO-ESN	50	100	0.1	0.01	50	0.3	0.01

**Table 3:** Prediction performance of six models for datasets MG(1) and MG(2); number of running times is 50.

	Models	RMSE		RMAE		MAPE		RMSPE		DA		Performance Order
		Mean Value	Standard Deviation	Mean Value	Standard Deviation	Mean Value	Standard Deviation	Mean Value	Standard Deviation	Mean Value	Standard Deviation	
MG(1)	ELM	4.2333 × 10^−4^	3.6354 × 10^−5^	2.7920 × 10^−4^	1.6822 × 10^−5^	3.5139 × 10^−4^	2.7389 × 10^−5^	6.6799 × 10^−4^	9.5655 × 10^−5^	100.00%	0	3
	PSO-ELM	4.0988 × 10^−5^	1.3745 × 10^−5^	2.1236 × 10^−5^	5.6891 × 10^−6^	2.5438 × 10^−5^	8.2240 × 10^−6^	5.8877 × 10^−5^	3.1339 × 10^−5^	100.00%	0	2
	EN-PSO-ELM	3.3164 × 10^−5^	1.3182 × 10^−5^	1.5342 × 10^−5^	5.4203 × 10^−6^	2.1275 × 10^−5^	8.1346 × 10^−6^	6.4802 × 10^−5^	3.1238 × 10^−5^	100.00%	0	1
	ESN	2.0595 × 10^−3^	7.0927 × 10^−4^	1.3627 × 10^−3^	5.0073 × 10^−4^	1.6014 × 10^−3^	5.7275 × 10^−4^	2.9686 × 10^−3^	1.0756 × 10^−3^	99.0201%	5.1431 × 10^−3^	3
	PSO-ESN	2.0336 × 10^−3^	4.7435 × 10^−4^	1.2950 × 10^−3^	3.6885 × 10^−4^	1.5015 × 10^−3^	4.4408 × 10^−4^	2.7100 × 10^−3^	8.3517 × 10^−4^	98.9950%	7.2647 × 10^−3^	2
	EN-PSO-ESN	1.0973 × 10^−3^	4.6523 × 10^−4^	9.7777 × 10^−4^	3.5712 × 10^−4^	1.0609 × 10^−3^	4.2366 × 10^−4^	1.3206 × 10^−3^	7.8256 × 10^−4^	98.9950%	5.3627 × 10^−3^	1
MG(2)	ELM	3.7327 × 10^−4^	1.8502 × 10^−5^	3.0227 × 10^−4^	1.3882 × 10^−5^	4.0961 × 10^−4^	2.1957 × 10^−5^	6.6574 × 10^−4^	5.1463 × 10^−5^	100.00%	0	3
	PSO-ELM	2.9236 × 10^−5^	6.7239 × 10^−6^	2.3141 × 10^−5^	5.2118 × 10^−6^	2.8240 × 10^−5^	7.4557 × 10^−6^	4.3585 × 10^−5^	2.2779 × 10^−5^	100.00%	0	1
	EN-PSO-ELM	3.4774 × 10^−5^	6.6342 × 10^−6^	2.7965 × 10^−5^	5.0656 × 10^−6^	3.7718 × 10^−5^	7.3756 × 10^−6^	7.3590 × 10^−5^	2.0232 × 10^−5^	100.00%	0	2
	ESN	3.7092 × 10^−2^	2.6262 × 10^−2^	2.9828 × 10^−2^	1.9397 × 10^−2^	3.1434 × 10^−2^	1.9213 × 10^−2^	4.3163 × 10^−2^	2.8004 × 10^−2^	76.8342%	7.7177 × 10^−2^	3
	PSO-ESN	1.7372 × 10^−2^	7.2545 × 10^−3^	1.5625 × 10^−2^	6.6169 × 10^−3^	1.5896 × 10^−2^	8.3831 × 10^−3^	1.9330 × 10^−2^	1.1820 × 10^−3^	79.8995%	1.3707 × 10^−2^	2
	EN-PSO-ESN	2.6844 × 10^−3^	7.2331 × 10^−3^	2.2819 × 10^−3^	6.4356 × 10^−3^	2.7199 × 10^−3^	8.2356 × 10^−3^	4.0754 × 10^−3^	1.0298 × 10^−4^	97.9899%	1.1635 × 10^−2^	1

**Table 4:** Prediction performance of six models for NARMA time series with *k* = 6 and *k* = 10; number of running times is 50.

	Models	RMSE		RMAE		MAPE		RMSPE		DA		Performance Order
		Mean Value	Standard Deviation	Mean Value	Standard Deviation	Mean Value	Standard Deviation	Mean Value	Standard Deviation	Mean Value	Standard Deviation	
NARMA(1)	ELM	8.4527 × 10^−2^	1.5725 × 10^−3^	2.0259 × 10^−1^	4.2477 × 10^−3^	1.9763 × 10^−1^	4.2896 × 10^−3^	2.4655 × 10^−1^	5.0867 × 10^−3^	68.1658%	1.7849 × 10^−2^	2
	PSO-ELM	8.7526 × 10^−2^	3.0443 × 10^−3^	2.1204 × 10^−1^	7.0243 × 10^−3^	2.1022 × 10^−1^	6.5851 × 10^−3^	2.6206 × 10^−1^	8.3883 × 10^−3^	66.3317%	1.5415 × 10^−2^	3
	EN-PSO-ELM	8.3184 × 10^−2^	2.6577 × 10^−3^	2.0105 × 10^−1^	6.2344 × 10^−3^	1.9733 × 10^−1^	5.8975 × 10^−3^	2.4445 × 10^−1^	7.5654 × 10^−3^	71.3568%	1.1103 × 10^−2^	1
	ESN	1.3926	3.6549 × 10^−1^	3.3487	7.6720 × 10^−1^	3.6671	9.0628 × 10^−1^	5.0313	1.4765	51.2563%	2.3409 × 10^−2^	3
	PSO-ESN	2.5375 × 10^−1^	1.6762 × 10^−2^	6.5980 × 10^−1^	5.2713 × 10^−2^	7.1371 × 10^−1^	7.0387 × 10^−2^	9.0056 × 10^−1^	7.8975 × 10^−2^	57.2864%	1.3531 × 10^−2^	2
	EN-PSO-ESN	8.8658 × 10^−2^	1.4525 × 10^−2^	2.2177 × 10^−1^	4.7286 × 10^−2^	2.2927 × 10^−1^	6.6895 × 10^−2^	2.8936 × 10^−1^	6.5657 × 10^−2^	68.8442%	1.0126 × 10^−2^	1
NARMA(2)	ELM	7.5817 × 10^−2^	1.8216 × 10^−3^	1.6056 × 10^−1^	2.9249 × 10^−3^	1.6436 × 10^−1^	2.6978 × 10^−3^	2.0151 × 10^−1^	4.9365 × 10^−3^	65.5276%	1.9358 × 10^−2^	2
	PSO-ELM	7.9777 × 10^−2^	1.7392 × 10^−3^	1.7079 × 10^−1^	3.6645 × 10^−3^	1.7622 × 10^−1^	4.4762 × 10^−3^	2.1918 × 10^−1^	6.4204 × 10^−3^	65.3266%	2.0750 × 10^−2^	3
	EN-PSO-ELM	7.6952 × 10^−2^	1.2366 × 10^−3^	1.6418 × 10^−1^	3.0012 × 10^−3^	1.6772 × 10^−1^	3.8745 × 10^−3^	2.0773 × 10^−1^	4.8673 × 10^−3^	67.8392%	1.5266 × 10^−2^	1
	ESN	9.1040 × 10^−1^	2.2495 × 10^−1^	1.8900	3.9288 × 10^−1^	2.1331	4.7139 × 10^−1^	2.9487	7.9041 × 10^−1^	51.9598%	2.5594 × 10^−2^	3
	PSO-ESN	2.1788 × 10^−1^	2.5335 × 10^−3^	4.4598 × 10^−1^	6.2503 × 10^−3^	4.9090 × 10^−1^	7.9616 × 10^−3^	6.6947 × 10^−1^	9.9815 × 10^−3^	56.7839%	2.0675 × 10^−2^	2
	EN-PSO-ESN	7.5906 × 10^−2^	2.4659 × 10^−3^	1.5905 × 10^−1^	6.0325 × 10^−3^	1.6497 × 10^−1^	7.2377 × 10^−3^	2.1019 × 10^−1^	9.3257 × 10^−3^	67.3367%	1.6885 × 10^−2^	1

**Table 5:** Prediction performance of six models for Henon attractor and Lorenz attractor; number of running times is 50.

	Models	RMSE		RMAE		MAPE		RMSPE		DA		Performance Order
		Mean Value	Standard Deviation	Mean Value	Standard Deviation	Mean Value	Standard Deviation	Mean Value	Standard Deviation	Mean Value	Standard Deviation	
Henon attractor	ELM	3.3204 × 10^−6^	1.9751 × 10^−6^	4.4166 × 10^−5^	2.6936 × 10^−5^	3.7655 × 10^−5^	2.3881 × 10^−5^	1.5742 × 10^−4^	1.0435 × 10^−4^	100.00%	0	3
	PSO-ELM	1.4408 × 10^−6^	1.7335 × 10^−6^	1.8179 × 10^−5^	2.4365 × 10^−5^	1.7493 × 10^−5^	1.4665 × 10^−5^	8.0322 × 10^−5^	3.2457 × 10^−5^	100.00%	0	2
	EN-PSO-ELM	6.8053 × 10^−7^	1.6568 × 10^−6^	8.0781 × 10^−6^	2.4415 × 10^−6^	8.3876 × 10^−6^	1.3622 × 10^−5^	4.3527 × 10^−5^	3.1757 × 10^−5^	100.00%	0	1
	ESN	1.2784 × 10^−5^	5.6772 × 10^−6^	1.2730 × 10^−4^	5.3143 × 10^−5^	1.3102 × 10^−4^	5.8792 × 10^−5^	8.0622 × 10^−4^	5.8563 × 10^−4^	100.00%	0	3
	PSO-ESN	4.0724 × 10^−6^	1.9122 × 10^−7^	4.4212 × 10^−5^	1.9947 × 10^−6^	6.4647 × 10^−5^	2.1198 × 10^−6^	5.6427 × 10^−4^	1.3948 × 10^−5^	100.00%	0	2
	EN-PSO-ESN	9.4210 × 10^−8^	1.8652 × 10^−7^	9.2122 × 10^−7^	1.8257 × 10^−6^	1.1861 × 10^−6^	1.9121 × 10^−6^	9.5494 × 10^−6^	1.2398 × 10^−5^	100.00%	0	1
Lorenz attractor	ELM	8.9132 × 10^−5^	6.7269 × 10^−5^	3.9429 × 10^−6^	2.9819 × 10^−6^	4.6187 × 10^−6^	3.4981 × 10^−6^	5.0824 × 10^−6^	3.8467 × 10^−6^	100.00%	0	3
	PSO-ELM	4.4364 × 10^−5^	3.0233 × 10^−5^	1.1664 × 10^−6^	1.0605 × 10^−6^	1.3840 × 10^−6^	1.2885 × 10^−6^	2.4607 × 10^−6^	2.0413 × 10^−6^	100.00%	0	2
	EN-PSO-ELM	1.5507 × 10^−5^	2.9626 × 10^−5^	4.9162 × 10^−7^	9.2377 × 10^−7^	4.9223 × 10^−7^	1.0132 × 10^−6^	6.4194 × 10^−7^	1.8652 × 10^−6^	100.00%	0	1
	ESN	9.0528 × 10^−3^	7.2441 × 10^−3^	2.5788 × 10^−4^	2.1277 × 10^−4^	3.9498 × 10^−4^	3.5720 × 10^−4^	7.2311 × 10^−4^	6.5796 × 10^−4^	99.6315%	4.2861 × 10^−3^	3
	PSO-ESN	8.0337 × 10^−3^	2.3815 × 10^−3^	9.6566 × 10^−5^	6.0522 × 10^−5^	2.0888 × 10^−4^	6.1014 × 10^−4^	7.5513 × 10^−4^	1.0421 × 10^−4^	98.4925%	1.1511 × 10^−3^	2
	EN-PSO-ESN	6.4638 × 10^−4^	2.2356 × 10^−3^	2.4464 × 10^−5^	5.8677 × 10^−5^	2.6527 × 10^−5^	5.8206 × 10^−4^	3.1241 × 10^−5^	8.9885 × 10^−5^	100.00%	0	1

**Table 6:** Prediction performance of six models (AQ dataset); number of running times is 50.

	Models	RMSE		RMAE		MAPE		RMSPE		DA		Performance Order
		Mean Value	Standard Deviation	Mean Value	Standard Deviation	Mean Value	Standard Deviation	Mean Value	Standard Deviation	Mean Value	Standard Deviation	
AQ	ELM	6.0336 × 10^−1^	3.1730 × 10^−2^	2.0693 × 10^−1^	1.0420 × 10^−2^	3.1946 × 10^−1^	1.5002 × 10^−2^	9.2986 × 10^−1^	7.0943 × 10^−2^	82.3036%	1.3441 × 10^−2^	3
	PSO-ELM	5.1643 × 10^−1^	2.5506 × 10^−2^	1.8407 × 10^−1^	1.0414 × 10^−2^	3.0602 × 10^−1^	2.3974 × 10^−2^	8.9843 × 10^−1^	9.2220 × 10^−2^	81.3022%	1.7108 × 10^−2^	2
	EN-PSO-ELM	5.1077 × 10^−1^	2.2681 × 10^−2^	1.7526 × 10^−1^	1.0287 × 10^−2^	2.9757 × 10^−1^	2.0834 × 10^−2^	8.6287 × 10^−1^	8.9669 × 10^−2^	84.2763%	1.4262 × 10^−2^	1
	ESN	5.2005 × 10^−1^	6.1308 × 10^−1^	1.8365 × 10^−1^	8.0975 × 10^−2^	4.0563 × 10^−1^	1.1006 × 10^−1^	1.8808	4.5650 × 10^−1^	83.6772%	2.0616 × 10^−2^	3
	PSO-ESN	4.7754 × 10^−1^	2.0790 × 10^−2^	1.5994 × 10^−1^	8.6829 × 10^−3^	3.9858 × 10^−1^	2.5555 × 10^−2^	1.3818	1.0542 × 10^−1^	86.2597%	1.1972 × 10^−2^	2
	EN-PSO-ESN	4.5206 × 10^−1^	1.8512 × 10^−2^	1.5012 × 10^−1^	7.8701 × 10^−3^	3.7002 × 10^−1^	2.3122 × 10^−2^	1.2874	8.2653 × 10^−2^	88.0127%	8.2701 × 10^−3^	1

**Table 7:** Prediction performance of six models (ASN dataset); number of running times is 50.

	Models	RMSE		RMAE		MAPE		RMSPE		DA		Performance Order
		Mean Value	Standard Deviation	Mean Value	Standard Deviation	Mean Value	Standard Deviation	Mean Value	Standard Deviation	Mean Value	Standard Deviation	
ASN	ELM	5.2713	4.7943 × 10^−1^	3.2036 × 10^−2^	2.4332 × 10^−3^	3.1748 × 10^−2^	2.4495 × 10^−3^	4.1038 × 10^−2^	3.7342 × 10^−3^	61.0726%	3.3335 × 10^−2^	2
	PSO-ELM	5.3758	1.0927	3.2501 × 10^−2^	6.0878 × 10^−3^	3.2192 × 10^−2^	6.3756 × 10^−3^	4. 1941 × 10−^2^	9.3557 × 10^−3^	59.9668%	3.4171 × 10^−2^	3
	EN-PSO-ELM	5.2748	9.0837 × 10^−1^	3.0147 × 10^−2^	5.7950 × 10^−3^	3.0231 × 10^−2^	6.2611 × 10^−3^	4.1590 × 10^−2^	8.9755 × 10^−3^	69.0036%	3.2435 × 10^−2^	1
	ESN	8.6583	4.3130 × 10^−1^	4.8673 × 10^−2^	9.7585 × 10^−2^	4.8916 × 10^−2^	3.8668 × 10^−2^	6.9217 × 10^−2^	3.5095 × 10^−2^	61.8763%	3.4994 × 10^−2^	3
	PSO-ESN	5.6447	5.5497 × 10^−1^	3.3215 × 10^−2^	3.3487 × 10^−3^	3.2834 × 10^−2^	3.4597 × 10^−3^	4.4033 × 10^−2^	4.5414 × 10^−3^	64.1582%	4.2528 × 10^−2^	2
	EN-PSO-ESN	5.0717	5.3557 × 10^−1^	2.9766 × 10^−2^	3.1642 × 10^−3^	2.9418 × 10^−2^	3.2883 × 10^−3^	3.9145 × 10^−2^	4.3776 × 10^−3^	65.0027%	3.0217 × 10^−2^	1

**Table 8:** Models compared in additional experiment.

		Description
Models	Canonical ELM model	The traditional ELM model without pre-training
	PSO-ELM(I) model	Part of the non-zero elements in the ***W^in^*** will be selected as a candidate for pre-training by PSO
	PSO-ELM(II) model	Part of the element in the ***W^in^***, including non-zero and zero elements, will be selected as candidates for pre-training by PSO
	EN-PSO-ELM model	Ensemble strategy combined with PSO-ELM(II) model

## Data Availability

Data are contained within the article.
